# The role side effects play in the choice of antiepileptic therapy in brain tumor-related epilepsy: a comparative study on traditional antiepileptic drugs versus oxcarbazepine

**DOI:** 10.1186/1756-9966-28-60

**Published:** 2009-05-06

**Authors:** Marta Maschio, Loredana Dinapoli, Antonello Vidiri, Andrea Pace, Alessandra Fabi, Alfredo Pompili, Maria Carmine Carapella, Bruno Jandolo

**Affiliations:** 1 Center for tumor-related Epilepsy, Department of Neuroscience and Cervical- Facial Pathology, National Institute for Cancer "Regina Elena", Via Elio Chianesi 53, 00144 Roma, Italy; 2 Department of Radiology, National Institute for Cancer "Regina Elena", Via Elio Chianesi 53, 00144 Roma, Italy; 3 Department of Neuroscience and Cervical-Facial Pathology, National Institute for Cancer "Regina Elena", Via Elio Chianesi 53, 00144 Roma, Italy; 4 Division of Clinical Oncology, National Institute for Cancer "Regina Elena", Via Elio Chianesi 53, 00144 Roma, Italy

## Abstract

**Background:**

Seizure control doesn't represent the only challenging goal in patients with brain tumor-related epilepsy. Side effects have often taken precedence for patients' quality of life.

**Methods:**

We performed an observational retrospective study on patients with brain tumor-related epilepsy: 35 who had assumed oxcarbazepine monotherapy and 35 patients who had undergone treatment with traditional antiepileptic drugs. Primary variable of efficacy was the mean seizure frequency per month and safety variables were the drop-out for side effects and total incidence of side effects. We applied the Propensity Score technique to minimize selection bias.

**Results:**

Our results showed a similar efficacy of oxcarbazepine and traditional antiepileptic drugs over time, but the difference in safety and tolerability between the two groups was significant: traditional AEDs caused more side effects, both serious and non serious.

**Conclusion:**

This study highlights the importance of taking into consideration not only seizure control but also the appearance of side effects when choosing antiepileptic drugs in this patients population.

## Background

Seizures are a common symptom in patients with brain tumors [1]. Literature data on antiepileptic drugs (AEDs) in brain tumor patients indicate that not only complete seizure control is a challenging goal [2] but that reducing unpleasant side effects produced by AEDs is a serious concern as well [3]. Side effects are mostly associated with the administration of traditional, older antiepileptic drugs: carbamazepine (CBZ), phenobarbital (PB), phenytoin (PHT) and valproic acid (VPA) [3-7]. Some limited data in the literature indicate that side effects are less marked when the newer AEDs such as oxcarbazepine, levetiracetam, topiramate, gabapentin and pregabalin are administered [6-13]. However, there have been no comparative studies to date which document the differences in efficacy and tolerability between the newer and older AEDs. The aim of this study was to assess if one of the newer generation AEDs presented significant differences in terms of efficacy as well as safety/tolerability when compared to the traditional AEDs, in patients with brain-tumor related epilepsy.

We chose not to undertake a comparative prospective study using traditional AEDs versus new AEDs, because substantial data indicate high toxicity of traditional AEDs and their interactions with chemotherapeutic agents strong enough to shorten life expectancy [7,14-18]. Therefore, we preferred to compare two retrospective groups, one in therapy with traditional AEDs and one with a new generation AED – oxcarbazepine – in order to assess if there were differences in efficacy and tolerability.

We choose a retrospective group of patients treated with oxcarbazepine because its efficacy is similar to that observed with the old AEDs [19], but, the low induction of CYP enzymes by OXC is associated with lower pharmacological interaction than other drugs. For this reason, also the interactions with chemotherapeutics agents appear unlikely [20,21].

## Methods

### Study design

We made a retrospective chart review for 35 brain tumor patients who were followed in our Institute because of brain tumor and epilepsy during the period 1995 to December 2005 (the last date which had been documented in the medical charts). These patients had been in treatment with traditional AEDs (*Traditional AEDs group*). We chose those patients whose age, sex and duration of AED treatment were similar to the OXC group.

We conducted a retrospective chart review on 35 patients with brain tumor and epilepsy who came to our Center during the period January, 2002 to February, 2007 in order to evaluate the efficacy and tolerability of OXC monotherapy (*OXC group*). Data were collected from medical charts until June 2007 (data chosen for the end of the study).

We compared the Traditional AED group to the OXC group in order to assess if there were differences in efficacy and tolerability.

The study was approved by the Institute's Ethical Committee.

### Selection of patients

Patients with brain tumor related epilepsy were included in the study if: between the ages 18 and 85; if they had had a KPS ≥ 60; if they had received a diagnosis of their disease (primary brain tumors or metastatic brain tumors) after surgical intervention or radiological diagnosis. Patients were eligible for inclusion if they had experienced at least one observable seizure in the last year, prior to screening. Patients with epilepsy unrelated to brain tumor were excluded from the study. The following information was collected for each patient, at baseline and during the history of disease: surgery, type of chemotherapy, radiotherapy, presence of a tumoral progression.

### Assessment methods

#### Traditional AED group and OXC group

A retrospective chart review was conducted on 35 brain tumor patients who had received PB, CBZ, PHT or VPA monotherapy for seizure control and on 35 brain tumor patients who had received OXC monotherapy for seizure control at our Center. These patients had arrived at our Center: 1) for uncontrolled seizures and/or side effects which had been caused by previous AED therapy 2) soon after the diagnosis of epilepsy related to brain tumor, without having had any prior AED therapy. Seizure frequency (SF) was assessed based on number of seizures documented in patient histories, hospital charts, and clinic notes. The appearance of side effects was assessed by using clinical notes and hospital charts. The severity of the AED's side effects was evaluated using the "Common Terminology Criteria for Adverse Events" [22].

### Statistical analyses

The aim of the study was to conduct a comparative analysis between the treatment groups: A) *OXC Group *and B) *Traditional AED Group *in order to evaluate the efficacy in controlling seizures as well as the safety and tolerability of the AEDs. The primary efficacy variable which we used was the mean number of seizures per month. The safety variables used were both the drop-out for side effects as well as the total incidence of side effects.

In order to subject our data to statistical analyses, it was necessary to create homogeneity between the two treatment groups (OXC and Traditional AEDs). This was done by applying a Propensity Score (PS), even though this technique is primarily used for larger samples. A PS is used by identifying co-variables in both groups to insert in the logistic regression model. Seven co-variables useful for the analysis were identified: age, sex, tumor progression, KPS, chemotherapy, seizure frequency at base visit, follow-up duration. The statistical analysis of efficacy between treatment groups was applied using a General Linear Model for fixed factors (GLM), taking into consideration the following factors: 1) Treatment Group (OXC versus Traditional AEDs) 2) Visit (baseline versus final follow-up) 3) Interaction between Treatment Group and Visit. The PS was applied only for the analysis of efficacy between treatment groups, and not for the safety/tolerability comparison between groups. For the analysis of safety variables (drop-out incidence and total incidence of side effects) we used the Fisher Exact Test taking into consideration the number of patients who had left the study or who had had side-effects.

The changes of SF from baseline to the final follow-up visit were evaluated using statistical analysis on the intent-to-treat (ITT) population (that is patients who had had at least one on-treatment visit with seizure counts).

## Results

### Traditional AED group

#### Patient Profiles

Patients' demographic and clinical characteristic are depicted in table 1 [see additional file 1].

Sixteen (16) had had glioblastoma multiforme (GBM), 5 anaplastic astrocytoma (AA), 4 anaplastic oligodendroglioma (AO), 8 low grade astrocytoma (LGA) and 2 low grade oligodendroglioma (LGO). Fourteen patients had undergone only chemotherapy during the follow up, 7 patient had undergone only radiotherapy, 11 chemotherapy and radiotherapy and 3 patients had not undergone any systemic therapy. Eight patients had had tumoral progression during follow up.

The mean age at diagnosis of brain tumor was 50.1 years (range 22 to 76 years). Nine patient had had simple partial seizures (SP), 9 had had complex partial (CP), 3 had had SP + secondarily generalized tonic clonic seizures (SP+SGTC) and 14 had had CP+SGTC seizures. Patients had all been in monotherapy with traditional AEDs: PB (N = 24); CBZ (N = 9); VPA (N = 1), PHT (N = 1). Mean dosages: PB = 112.5 mg/day, CBZ = 800 mg/day, VPA 1000 mg/day (only 1 patient), PHT 200 mg/day (only 1 patient) [see additional file 1].

#### Efficacy

The mean seizure frequency per month before AED treatment had been 4.1 (35 patients) and 1.6 (35 patients) at final follow up. At final follow up, 45.7% of patients (16 patients) were seizure free. GLM repeated measure analysis showed a significant reduction of seizure frequency at final follow-up (p = 0.0095). Mean duration of follow up was 13.7 months (range 2 to 48 months).

#### Adverse Events

During treatment fifteen patients (42.9%) had reported side effects: 11 patients in therapy with PB, 3 with CBZ and 1 with VPA. Two patients (5.7%), all in therapy with CBZ, had had mild and reversible side effects (haematological toxicity) and 13 patient (37.2%) had had heavy side effects: 5 psychomotor slowness (4 patients with PB and 1 with VPA), 4 rash (all patient with PB), 2 periarthritis (all patients with PB), 1 somnolence (patient with PB) and 1 liver toxicity (patient with CBZ) [See additional file 2].

### OXC Group

#### Patient Profiles

Patients' demographic and clinical characteristic are depicted in table 3 [see additional file 3].

Twelve patients had brain metastases, 4 GBM, 10 AA, 1 OA, 6 LGA and 2 meningioma. During follow up, 6 patients had undergone only chemotherapy, 3 patients had undergone only radiotherapy, 23 patients had undergone both chemotherapy and radiotherapy and 3 patients had not undergone any systemic therapy. Fourteen patients had had tumoral progression. The mean age at diagnosis of brain tumor was 52 years (range 18 to 81 years).

Eleven patients had had SP seizures, 4 had had CP, 6 had had SP+SGTC and 14 had had CP+SGTC seizures.

Eighteen patients had already been treated with other AEDs: PB = 14; CBZ = 3; topiramate – TPM- (N = 1) that had been changed to OXC for heavy side effects (8 patients), uncontrolled seizures (9 patients) and 1 for uncontrolled seizures and heavy side effects. Mean dosages had been: PB = 103.6 mg/day, CBZ = 466.70 mg/day, TPM 150 (only 1 patient). Seventeen had been naïve patients. During the period considered for the study, patients had all been in monotherapy with OXC with a mean daily dosage of 1162.5 mg [See additional file 4].

### Efficacy

The mean seizure frequency per month before OXC therapy had been 2.9, and at the final follow-up had been 0.6 (35 patients). Considering separately the two subgroups naive patients versus patients presenting for side effects/inefficacy, the mean seizure frequency per month before OXC therapy had been 4.64 (naïve patients, 17 patients) and 1.3 (non-naïve patients, 18 patients). At the final follow-up the mean seizure frequency had been 0.88 (naïve patients) and 0.4 (non-naïve patients). At final follow up, we obtained 62.9% patients who were seizure free (22 patients). GLM repeated measure analysis showed a significant reduction of seizure frequency at final follow-up (p = 0.0018). Mean duration of follow up was 16.1 months (range 4 to 48 months).

#### Adverse Events

During follow up 4 patients (11.4%) reported side effects: 1 patient (2.9%) had had mild and reversible side effects (mild rash and liver toxicity) and 3 (8.6%) had had heavy side effects (2 rash and 1 cephalea) [See additional file 4].

### Comparison between the two groups

#### Efficacy

In order to compare monthly seizure frequency in both groups we used GLM repeated measure analysis with variables: treatment groups (Traditional AEDs versus OXC group), visit (baseline versus follow up), and interaction Group × Visit. Statistical analysis for both groups showed a significant reduction of seizure frequency between first visit and last follow up visit (p < 0.0001). The comparison made between treatment groups and interaction Group × Visit is not significant (p = 0.1166 between groups; p = 0.9221 Group × Visit).

#### Adverse Events

Taking into consideration the first variable of safety, drop out for side effects, the Fisher exact test showed a significant difference between the OXC group and the Traditional AED group (p = 0.0090)(Odds ratio = 6.303). In particular, concerning drop-out due to heavy side effects, only 3 patients in the OXC group and 13 patients of Traditional AEDs group were forced to stopped the AEDs.

Taking into consideration the second variable of safety, total incidence of side effects, Fisher exact test showed a significant difference between the OXC group and the Traditional AED group (p = 0.0063)(Odds ratio = 5.813). In particular, four patients had side effects during OXC treatment whereas 15 patients in the Traditional AEDs group had side effects.

## Discussion

Epilepsy is considered the most important risk factor for long-term disability in brain tumour patients [23]. Unfortunately, the side effects related to antiepileptic drugs can seriously affect the patients' quality of life; in fact, it has been found that patients' concerns with the AEDs' side effects have often taken precedence over their desire to reduce seizure frequency [24]. Side effects are mostly associated with the administration of traditional, older AEDs [3-8]. The few studies which have been done on the newer AEDs indicate that these same side effects are less frequent with these drug [9-13]. To date, a comparative study of this type has not been done.

We performed a statistical analysis and applied a Propensity Score in order to minimize the selection bias and other sources of bias. Concerning efficacy, results showed no major differences between the two groups. Concerning safety and tolerability, however, the profiles differ significantly. The traditional AED group had had more side effects than the OXC group (42.9% vs 11.4%), including heavy side effects which led patients to discontinue usage of the AED. It is generally accepted that the percentage of patients withdrawing because of adverse effects represents a reliable marker of tolerability [25].

The percentage of side effects for OXC was similar to that observed in non-tumoral, epileptic patients (10%)[19], and the percentage of side effects for traditional AEDs is consistent with literature data (5 to 38% in patients with brain tumor-related epilepsy)[3].

The most common side effects we found were rash (11.4% in Traditional AEDs group and 8.6% in OXC group) and psychomotor slowness (21.7% only in Traditional AEDs group). In epileptic, non-tumoral patients, rash is a common side effect associated with most AED use, ranging between 3–10% and has been the leading cause of withdrawal from some AED trials [6,26]. The available data to date indicate that in patients with brain tumor-related epilepsy, the incidence of severe rash is higher than in non-tumoral, epileptic patients (14%)[3]. This is in part due to the fact that patients with brain tumor often undergo radiotherapy and chemotherapy and this association induces an increased incidence of skin reactions in patients assuming old AEDs like PHT, CBZ and PB [4]. Our results are consistent with these literature data. Regarding psychomotor slowness our results are consistent with literature data that shows that patients with brain tumor-related epilepsy taking CBZ, VPA, PB and PHT performed worse in all cognitive domains than patients who did not undergo any AED therapy [6]. It is important to note that literature data cites cognitive impairment in brain tumor patients as much more common than the physical disability [27,28]. Such impairment is the major variable which influences quality of life in patients with epilepsy [29]. For this reason, the choice of an AED which does not impair cognitive functioning is of primary importance for patients with brain tumor-related epilepsy.

Concerning efficacy, we observed a similarly good profile of efficacy over time in the two groups of treatment, with a significant reduction in number of seizures. However, the comparison between treatment groups is not significant. Studies to date dedicated specifically to the efficacy of the new AEDs in controlling seizures in patients with brain tumor-related epilepsy, are very recent [9-12].

In the literature only one study examined OXC monotherapy only in patients with brain tumor-related epilepsy [11]. This study was conducted for preventing perioperative seizures in patients with brain tumors. In the other studies, OXC is one of many drugs tested [14,15,30]. Recently, one study was done using OXC monotherapy in patients with cryptogenetic or symptomatic epilepsy [31]. In this study the efficacy of OXC is significantly more pronounced in patients with cryptogenetic epilepsy than in patients with brain tumors. Our study is the first that uses only OXC in epilepsy related to brain tumor, with a long-term follow up and with a good efficacy.

With regard to follow-up, it is important to point out the difficulty that the death of patients poses in studies of patients with this type of cancer. It should be noted that the mortality rate of patients with brain tumors makes long-term studies difficult and presents problems already at the onset with obtaining a significant number of participants for studies. In the two groups, the follow up varied from 2 to 48 months: this variability is due to deceased patients. This has already been mentioned as being a serious drawback to studies on this patient population.

In our study, both groups of patients were in treatment with chemotherapy, and data in the literature indicate that chemotherapy could play a role in seizure control [32]. Therefore, the fact that systemic therapy might have affected the outcome cannot be excluded.

In our study we have patients with different histological diagnosis, so we were unable to determine a difference in efficacy of AED therapy (traditional and OXC) in controlling seizures, based on the different histological diagnosis.

While we used the Propensity Score Technique to avoid selection bias, we cannot exclude the fact that data obtained in retrospective studies may affect the outcome concerning significant statistical differences in efficacy between the two groups.

## Conclusion

This is the first study which compares the older AEDs with a newer AED, in patients with brain tumor-related epilepsy. Our most significant findings concern the presence of side effects, both serious and less serious in patients who had assumed the older AEDs. It was the serious side effects which were largely present in the traditional AEDs group; the extent to which patients with these side effects were forced to interrupt treatment. This brings us to the issue of patients' quality of life, which we urge must take into consideration not only seizure control, but also adverse events; most studies to date focus primarily on the former and not the latter. Our study clearly demonstrates that while both traditional AEDs and oxcarbazepine may reduce seizure frequency equally as well, the higher incidence of serious side effects which make the traditional AEDs less tolerable, affect the quality of life of patients who must already face numerous drug therapies.

## Competing interests

The authors declare that they have no competing interests.

## Authors' contributions

MM conceived and conducted the study and wrote the paper. LD participated in study design and contributed to paper writing. JB participated in study coordination. VA performed patients radiological examination. PA, FA, PA and CMC collaborate to data acquisition. All authors read and approved the final manuscript.

## Supplementary Material

Additional file 1 **TRADITIONAL AEDs GROUP: Patients' clinical and vital data**. The data in table provide clinical and vital data of patients of traditional AEDs group. Click here for file

Additional file 2 **TRADITIONAL AEDs GROUP: Epilepsy characteristics**. The data in table provide epilepsy characteristics of patients of traditional AEDs group. Click here for file

Additional file 3 **OXC GROUP: Patients' clinical and vital data**. The data in table provide clinical and vital data of patients of OXC group. Click here for file

Additional file 4 **OXC GROUP: Epilepsy characteristics**. The data in table provide epilepsy characteristics of patients of OXC group. Click here for file
