# Supplementary material for: The role side effects play in the choice of antiepileptic therapy in brain tumor-related epilepsy: a comparative study on traditional antiepileptic drugs versus oxcarbazepine
Source: J Exp Clin Cancer Res. 2009 May 6;28(1):60. doi: 10.1186/1756-9966-28-60 (PMC2686682; doi:10.1186/1756-9966-28-60)
Supplement: Additional file 1 — TRADITIONAL AEDs GROUP: Patients' clinical and vital data. The data in table provide clinical and vital data of patients of traditional AEDs group. [file 1756-9966-28-60-S1.doc]

**Table 1 TRADITIONAL AEDs GROUP: Patients’ clinical and vital data.**

| **Patient** | **Age**  (years) | **Sex** | Histology | **Surgery** | **KPS at first**  **visit** | **Chemotherapy**  **at first visit** | **Chemotherapy**  ***** | Radiotherapyat first visit | Radiotherapy ***** | **Tumoral Progression**  ***** | Death |
| --- | --- | --- | --- | --- | --- | --- | --- | --- | --- | --- | --- |
| 1 | 35 | F | AO | PR | 100 | No | PCV | no | No | No | No |
| 2 | 66 | M | GBM | PR | 90 | No | PCV | no | Yes | Yes | Yes |
| 3 | 43 | F | AA | PR | 100 | No | TMZ | no | Yes | No | Yes |
| 4 | 66 | M | GBM | PR | 90 | No | TMZ | no | Yes | Yes | Yes |
| 5 | 26 | F | LGA | Biopsy | 100 | No | TMZ | no | No | No | Yes |
| 6 | 57 | F | AA | Biopsy | 100 | TMZ | PCV-TMZ | no | No | Yes | Yes |
| 7 | 50 | M | GBM | PR | 100 | No | PCV | no | No | No | Yes |
| 8 | 75 | M | LGA | Biopsy | 100 | No | TMZ | no | No | No | Yes |
| 9 | 43 | M | LGA | PR | 100 | No | TMZ | no | No | Yes | Yes |
| 10 | 76 | F | GBM | Biopsy | 90 | No | CCNU | no | No | Yes | Yes |
| 11 | 50 | M | GBM | PR | 90 | No | PCV | no | No | No | Yes |
| 12 | 55 | F | GBM | PR | 100 | TMZ | PCV | no | No | No | Yes |
| 13 | 38 | F | LGA | PR | 100 | No | No | no | Yes | No | Yes |
| 14 | 46 | M | AA | Biopsy | 90 | No | TMZ | no | No | No | Yes |
| 15 | 48 | M | AA | Biopsy | 90 | No | No | no | Yes | No | Yes |
| 16 | 51 | M | GBM | PR | 80 | No | TMZ | no | Yes | No | Yes |
| 17 | 64 | F | GBM | PR | 70 | PCV | PCV | no | Yes | No | Yes |
| 18 | 48 | F | AO | PR | 100 | No | No | no | Yes | No | No |
| 19 | 27 | M | LGO | Biopsy | 100 | No | PCV | no | No | No | No |
| 20 | 48 | M | AO | Biopsy | 100 | No | No | no | Yes | No | No |
| 21 | 39 | F | GBM | GTR | 100 | No | TMZ | no | Yes | Yes | No |
| 22 | 22 | F | LGA | GTR | 100 | No | No | no | No | No | Yes |
| 23 | 62 | M | GBM | GTR | 90 | No | PCV | no | Yes | No | Yes |
| 24 | 63 | M | GBM | PR | 90 | No | TMZ | no | Yes | No | Yes |
| 25 | 28 | F | LGA | GTR | 100 | No | TMZ | no | No | Yes | Yes |
| 26 | 35 | M | GBM | GTR | 100 | No | No | no | Yes | No | Yes |
| 27 | 57 | M | GBM | GTR | 100 | No | PCV-TMZ | no | Yes | No | Yes |
| 28 | 75 | M | AA | GTR | 100 | No | PCV-TMZ | no | No | Yes | Yes |
| 29 | 40 | M | LGO | GTR | 100 | No | No | no | No | No | No |
| 30 | 59 | F | LGA | PR | 100 | No | No | no | No | No | No |
| 31 | 48 | M | GBM | Biopsy | 80 | No | TMZ | no | Yes | No | Yes |
| 32 | 62 | M | LGA | Biopsy | 80 | No | No | no | Yes | No | Yes |
| 33 | 68 | G | GBM | GTR | 80 | No | TMZ | no | No | No | Yes |
| 34 | 61 | M | GBM | Biopsy | 80 | No | TMZ | no | Yes | No | Yes |
| 35 | 22 | M | AO | PR | 100 | No | No | no | Yes | No | No |

**Histological diagnosis**:

GBM, Glioblastoma Multiforme; AA, Anaplastic Astrocytoma; AA, Anaplastic Oligodendroglioma; LGA, Low Grade Astrocytoma; LGO, Low Grade Oligodendroglioma

**Chemotherapy**: TMZ, temozolomide; PCV, procarbazine, chloroethylnitrosourea and vincristine; CCNU, lomustine

***** = during the period of follow-up

**Surgery**: GTR, Gross Total Resection; PR, Partial Resection
