# Supplementary material for: The role side effects play in the choice of antiepileptic therapy in brain tumor-related epilepsy: a comparative study on traditional antiepileptic drugs versus oxcarbazepine
Source: J Exp Clin Cancer Res. 2009 May 6;28(1):60. doi: 10.1186/1756-9966-28-60 (PMC2686682; doi:10.1186/1756-9966-28-60)
Supplement: Additional file 2 — TRADITIONAL AEDs GROUP: Epilepsy characteristics. The data in table provide epilepsy characteristics of patients of traditional AEDs group. [file 1756-9966-28-60-S2.doc]

**Table 2 TRADITIONAL AEDs GROUP: Epilepsy characteristics**

| **Patient** | **Seizure types** | **AED therapy** (mg/day) | **SIDE EFFECTS** | **Monthly seizure frequency before AEDs therapy** | **Monthly seizure frequency**  **at last follow up** | **Duration of AEDs therapy** (months) | **Reasons for drop out** |
| --- | --- | --- | --- | --- | --- | --- | --- |
| 1 | CP+SGTC | PB 100 | Somnolence | 0.08 | 0 | 12 | Somnolence |
| 2 | SP+SGTC | PB 150 | - | 15 | 2 | 11 | - |
| 3 | CP | CBZ 800 | Haematological toxicity | 15 | 0 | 22 | No change |
| 4 | CP+SGTC | PHT 200 | - | 0.08 | 0 | 12 | - |
| 5 | CP | PB 100 | - | 2 | 0 | 11 | - |
| 6 | CP+SGTC | CBZ 800 | - | 0.8 | 0 | 24 | - |
| 7 | CP+SGTC | PB 100 | - | 0.8 | 2 | 12 | - |
| 8 | CP+SGTC | CBZ 600 | Haematological toxicity | 0.8 | 0.8 | 11 | No change |
| 9 | CP+SGTC | CBZ 1000 | - | 2 | 0.8 | 36 | Seizures |
| 10 | CP | PB 100 | - | 15 | 9 | 7 | - |
| 11 | CP+SGTC | PB 150 | - | 3 | 3 | 14 | Seizures |
| 12 | SP | PB 100 | Periarthritis | 0.8 | 3 | 12 | Periarthritis |
| 13 | SP | PB 100 | Periarthritis | 20 | 2 | 12 | Periarthritis |
| 14 | CP+SGTC | PB 100 | Rash | 0.08 | 0 | 5 | Rash |
| 15 | CP | PB 100 | Rash | 0.08 | 0 | 8 | Rash |
| 16 | CP+SGTC | CBZ 400 | - | 0.8 | 0 | 19 | - |
| 17 | SP | PB 100 | - | 2 | 2 | 6 | Seizures |
| 18 | CP+SGTC | PB 100 | Rash | 0.8 | 0 | 2 | Rash |
| 19 | SP | PB 100 | - | 9 | 9 | 4 | Seizures |
| 20 | SP+SGTC | PB 100 | Rash | 0.08 | 0 | 7 | Rash |
| 21 | SP | CBZ 1000 | - | 1 | 1 | 16 | - |
| 22 | CP | CBZ 600 | - | 1 | 9 | 8 | Seizures |
| 23 | CP | PB 200 | - | 2 | 2 | 8 | Seizures |
| 24 | SP | PB 100 | Psychomotor slowness | 9 | 0 | 12 | Psychomotor slowness |
| 25 | CP+SGTC | CBZ 800 | - | 2 | 0 | 48 | - |
| 26 | SP | CBZ 1200 | Hepatic toxicity | 15 | 0 | 15 | Liver toxicity |
| 27 | CP | PB 150 | - | 8 | 0.8 | 23 | - |
| 28 | CP+SGTC | PB 150 | - | 2 | 0.08 | 40 | - |
| 29 | CP+SGTC | VPA 1000 | Psychomotor slowness | 2 | 2 | 12 | Psychomotor slowness |
| 30  continued | SP | PB 100 | - | 1 | 1 | 9 | Seizures |
| 31 | CP | PB 100 | - | 1 | 1 | 5 | Seizures |
| 32 | SP | PB 100 | - | 7 | 7 | 2 | Seizures |
| 33 | CP | PB 100 | Psychomotor slowness | 2 | 0 | 3 | Psychomotor slowness |
| 34 | CP+SGTC | PB 100 | Psychomotor slowness | 2 | 0 | 28 | Psychomotor slowness |
| 35 | SP+SGTC | PB 100 | Psychomotor slowness | 2 | 0 | 3 | Psychomotor slowness |

**Seizures types**: SP, simple partial; CP, complex partial; SGTC, secondarily generalized tonic-clonic.

**Antiepileptic therapy**: PB, phenobarbital; CBZ, carbamazepine; VPA valproate; PHT, phenytoin
