# Supplementary material for: The role side effects play in the choice of antiepileptic therapy in brain tumor-related epilepsy: a comparative study on traditional antiepileptic drugs versus oxcarbazepine
Source: J Exp Clin Cancer Res. 2009 May 6;28(1):60. doi: 10.1186/1756-9966-28-60 (PMC2686682; doi:10.1186/1756-9966-28-60)
Supplement: Additional file 3 — OXC GROUP: Patients' clinical and vital data. The data in table provide clinical and vital data of patients of OXC group. [file 1756-9966-28-60-S3.doc]

**Table 3 OXC GROUP: Patients’ clinical and vital data.**

continued

| **Patient** | **Age** (years) | **Sex** | Histology | **Surgery** | **KPS at first visit** | **Chemotherapy at first visit** | **Chemotherapy *** | Radiotherapyat first visit | **Radiotherapy *** | **Total Survival Time** (months) | **Tumoral Progression *** | **Death** |
| --- | --- | --- | --- | --- | --- | --- | --- | --- | --- | --- | --- | --- |
| 1 | 54 | F | LGA | GTR | 80 | TMZ | TMZ | No | No | 21 | No | No |
| 2 | 32 | M | AA | GTR | 100 | No | TMZ | No | No | 7 | No | No |
| 3 | 39 | F | LGA | PR | 100 | PCV | TMZ,PCV,FTMU | No | No | 228 | Yes | Yes |
| 4 | 30 | F | AA | PR | 100 | No | TMZ,FTMU | No | No | 44 | Yes | Yes |
| 5 | 68 | F | MEN | No | 100 | No | No | No | No | 40 | No | No |
| 6 | 45 | M | AA | PR | 80 | TMZ | TMZ | No | No | 156 | Yes | Yes |
| 7 | 56 | M | MET (lung)° | No | 100 | No | Gefitinib | Stereotaxis | 3D-CRT | 18 | No | No |
| 8 | 35 | M | AA | GTR | 100 | No | TMZ | No | 3D-CRT | 18 | No | No |
| 9 | 39 | M | AA | PR | 100 | No | PCV | No | 3D-CRT | 36 | No | Yes |
| 10 | 49 | M | AA | PR | 90 | PCV | PCV | No | No | 16 | Yes | Yes |
| 11 | 81 | M | MET (lung)° | No | 80 | No | Gefitinib | Stereotaxis | No | 10 | No | Yes |
| 12 | 51 | F | MEN | GTR | 100 | No | No | No | No | 36 | No | No |
| 13 | 69 | M | GBM | GTR | 100 | TMZ | TMZ | No | 3D-CRT | 46 | Yes | No |
| 14 | 18 | F | LGA | GTR | 100 | No | No | No | No | 4 | No | No |
| 15 | 33 | M | AA | GTR | 100 | FTMU | FTMU | No | No | 65 | Yes | No |
| 16 | 62 | M | AA | PR | 100 | No | TMZ,FTMU | No | 3D-CRT | 16 | Yes | Yes |
| 17 | 78 | M | GBM | GTR | 80 | No | TMZ,FTMU | No | 3D-CRT | 26 | Yes | Yes |
| 18 | 45 | F | LGA | Biopsy | 90 | No | TMZ | No | Stereotaxis | 51 | Yes | No |
| 19 | 77 | M | GBM | GTR | 80 | TMZ | TMZ | No | No | 9 | No | Yes |
| 20 | 67 | F | MET (lung)° | No | 90 | DPP,GEM | DPP,GEM | Stereotaxis | No | 6 | No | Yes |
| 21 | 57 | F | AA | Biopsy | 70 | No | TMZ | No | 3D-CRT | 14 | No | No |
| 22 | 71 | M | MET (lung)° | No | 70 | CBDCA,GEM | CBDCA, GEM | No | WBRT | 6 | No | Yes |
| 23 | 37 | F | LGA | PR | 100 | VCR | PCV,TMZ,FTMU | No | No | 60 | Yes | Yes |
| 24 | 78 | M | MET (lung)° | No | 90 | No | No | No | Stereotaxis | 7 | No | Yes |
| 25 | 38 | F | LGA | PR | 100 | No | TMZ | No | No | 8 | No | No |
| 26 | 76 | M | GBM | GTR | 90 | No | TMZ | No | 3D-CRT | 4 | No | Yes |
| 27 | 44 | M | AO | PR | 100 | No | TMZ | 3D-CRT | No | 6 | No | No |
| 28 | 37 | F | AA | PR | 80 | TMZ | TMZ, FTMU | No | No | 18 | Yes | No |
| 29 | 35 | F | MET (breast)° | GTR | 100 | No | No | No | No | 20 | Yes | No |
| 30 | 47 | F | MET (breast) | No | 70 | No | VP-16 | No | WBRT | 6 | No | Yes |
| 31 | 68 | M | MET (lung)° | No | 80 | No | DDP GEM | WBRT | WBRT | 6 | No | No |
| 32 | 56 | M | MET (lung)° | No | 100 | No | DDP GEM | No | WBRT | 7 | No | Yes |
| 33 | 77 | F | MET (breast)° | GTR | 90 | No | Trastuzumab | No | No | 6 | No | No |
| 34 | 59 | M | MET (prostate)° | No | 100 | No | DDP+VP 16 | WBRT | WBRT | 5 | Yes | No |
| 35 | 43 | M | MET (melanoma)° | No | 80 | No | No | WBRT | WBRT | 6 | Yes | Yes |

**Histological diagnosis**: GBM, Glioblastoma Multiforme; AA, Anaplastic Astrocytoma; AO, Anaplastic Oligodendroglioma; LGA, Low Grade Astrocytoma; MEN, Meningioma, MET, brain metastasis

**Chemotherapy**: TMZ, temozolomide; PCV, procarbazine, chloroethylnitrosourea and vincristine; FTMU, fotemustine; VP16, etoposide; DDP, cisplatin; GEM, gemcitabin; CBDCA, carboplatin; VCR, vincristine

**Radiotherapy:** 3D-CRT, Three-Dimensional Conformational Radiation Therapy; WBRT**,** whole brain radiotherapy;

**Surgery**: GTR,Gross Total Resection; PR , Partial Resection

***** = during the period of follow-up

° = primitive tumor
