# Supplementary material for: The role side effects play in the choice of antiepileptic therapy in brain tumor-related epilepsy: a comparative study on traditional antiepileptic drugs versus oxcarbazepine
Source: J Exp Clin Cancer Res. 2009 May 6;28(1):60. doi: 10.1186/1756-9966-28-60 (PMC2686682; doi:10.1186/1756-9966-28-60)
Supplement: Additional file 4 — OXC GROUP: Epilepsy characteristics. The data in table provide epilepsy characteristics of patients of OXC group. [file 1756-9966-28-60-S4.doc]

**Table 4 OXC GROUP: Epilepsy characteristics**

| **Patient** | **Seizure types** | **AED therapy mg/day** (at study entry) | **OXC daily dose mg/day**  (at final follow-up) | **Reasons for introducing OXC** | **Seizure frequency at baseline** | **Seizure Frequency**  **at final follow-up** | **Time with OXC therapy** (months) |
| --- | --- | --- | --- | --- | --- | --- | --- |
| 1 | CP+SGTC | No | 900 | - | 2 | 0 | 23 |
| 2 | CP+SGTC | PB 100 | 1800 | Psychomotor slowness | 2 | 0 | 6  *Drop out Rash* |
| 3 | CP+SGCT | CBZ 400 | 1200 | Rash | 3 | 3 | 36 |
| 4 | SP | PB 50 | 1800 | Seizures | 3 | 1 | 48 |
| 5 | SP | No | 900 | - | 0.8 | 0 | 40 |
| 6 | CP | PB 100 | 1200 | Seizures | 0.8 | 0 | 8 |
| 7 | SP | No | 1200 | - | 3 | 0 | 18 |
| 8 | CP+SGTC | No | 1800 | - | 0.8 | 0.3 | 19 |
| 9 | CP+SGTC | PB 100 | 1200 | Periarthritis | 0.8 | 0 | 30 |
| 10 | CP+SGTC | PB 100 | 1500 | Seizures | 0.8 | 0 | 5 |
| 11 | SP | No | 1200 | - | 9 | 0 | 10 |
| 12 | CP+SGTC | No | 1500 | - | 9 | 4 | 36  *Drop out Rash* |
| 13 | CP+SGTC | PB 200 | 1800 | Seizures | 0.08 | 0.08 | 37 |
| 14 | CP+SGTC | No | 1200 | - | 0.8 | 0 | 4  *Drop Out Cephalea* |
| 15 | CP+SGTC | PB 100 | 1200 | Seizures | 0.8 | 0 | 42  *Mild rash hepatic toxicity* |
| 16 | CP+SGTC | PB 100 | 1200 | Periarthritis | 0.8 | 0 | 12 |
| 17 | SP | TPM 150 | 900 | Weight loss | 0.8 | 0 | 12 |
| 18 | SP+SGTC | CBZ 400 | 1200 | Seizures | 3 | 3 | 15 |
| 19 | CP | PB 100 | 1200 | Periarthritis | 2 | 0 | 5 |
| 20 | CP | No | 600 | - | 2 | 0 | 4 |
| 21 | SP | No | 1200 | - | 10 | 10 | 13 |
| 22 | CP+SGTC | No | 600 | - | 3 | 0 | 6 |
| 23 | CP+SGTC | CBZ 600 | 1200 | Rash | 0.08 | 0.08 | 48 |
| 24 | SP+SGTC | PB 100 | 900 | Seizures | 2 | 0 | 6 |
| 25 | SP+SGTC | No | 900 | - | 0.4 | 0 | 10 |
| 26 | SP | PB 100 | 900 | Seizures | 0.2 | 0 | 4 |
| 27 | SP | PB 100 | 900 | Rash | 3 | 0 | 4 |
| 28 | SP | No | 1200 |  | 0.16 | 0.16 | 13 |
| 29 | PS+SGTC | PB 100 | 1200 | Seizures | 0.25 | 0.1 | 8 |
| 30 | PC+SGTC | PB 100 | 1800 | Seizures/Somnolence | 0.08 | 0 | 7 |
| 31 | SP | No | 900 | - | 1 | 0.5 | 8 |
| 32 | SP+SGTC | No | 1200 | - | 2 | 0.02 | 7 |
| 33 | SP  continued | No | 900 | - | 4 | 0 | 7 |
| 34 | CP | No | 1200 | - | 30 | 0 | 6 |
| 35 | SP+SGTC | No | 1200 | - | 1 | 0.03 | 6 |

**Seizures types**: SP, simple partial; CP, complex partial; SGTC, secondarily generalized tonic-clonic.

**Antiepileptic therapy**: PB, phenobarbital; CBZ, carbamazepine; OXC, oxcarbazepine; TPM topiramate
